# Significant Enhanced Mechanical Properties of Suspended Graphene Film by Stacking Multilayer CVD Graphene Films

**DOI:** 10.3390/mi14040745

**Published:** 2023-03-28

**Authors:** Binbin Xiao, Mengqing Yin, Wanfa Li, Lingyan Liang, Shixun Dai, Xiaohui Zhang, Wei Wang, Zhaoping Liu

**Affiliations:** 1 Faculty of Electrical Engineering and Computer Science, Ningbo University, Ningbo 315211, China; 2 Key Laboratory of Graphene Technologies and Applications of Zhejiang Province, CAS Engineering Laboratory for Graphene, Ningbo Institute of Materials Technology & Engineering, Chinese Academy of Sciences, Ningbo 315201, China; 3 Key Laboratory of Advanced Nano Materials and Devices, Ningbo Institute of Materials Technology and Engineering, Chinese Academy of Sciences, Ningbo 315201, China; 4 CRRC Industrial Academy Co., Ltd., Beijing 100039, China

**Keywords:** chemical vapor deposition, suspended graphene film, mechanical properties

## Abstract

Suspended graphene film is of great significance for building high-performance electrical devices. However, fabricating large-area suspended graphene film with good mechanical properties is still a challenge, especially for the chemical vapor deposition (CVD)-grown graphene films. In this work, the mechanical properties of suspended CVD-grown graphene film are investigated systematically for the first time. It is found that monolayer graphene film is hard to maintain on circular holes with a diameter of tens of micrometers, which can be improved greatly by increasing the layer of graphene films. The mechanical properties of CVD-grown multilayer graphene films suspended on a circular hole with a diameter of 70 µm can be increased by 20%, and multilayer graphene films prepared by layer-layer stacking process can be increased by up to 400% for the same size. The corresponding mechanism was also discussed in detail, which might pave the way for building high-performance electrical devices based on high-strength suspended graphene film.

## 1. Introduction

Graphene’s extraordinary strength, excellent electrical properties, and good thermal conductivity make it of great research value and promising for a wide range of applications [1,2,3,4,5]. In most cases, the use of graphene film relies on supporting substrates, such as SiO_2_/Si, PET, etc. [6,7]. However, carrier scattering, doping, and phonon leakage induced by the substrate would lead to significant degradation of the intrinsic properties of graphene and hence result in graphene-based devices with performance below expectations [8]. Suspended graphene avoids direct contact between graphene and substrate, which would reach the intrinsic properties of the graphene without the influence of the substrate [9,10,11]. As a result, suspended graphene has attracted broad interest and shows great potential in fundamental research as well as commercial technologies [12,13,14]. For example, Lee et al. measured the mechanical properties of graphene film by transferring monolayer graphene onto silicon wafers with circular hole arrays to produce suspended graphene film [4]. Zhou et al. fabricated electrostatic graphene loudspeakers by transferring the multilayer graphene films grown on nickel foil to the prefabricated frame of the loudspeaker, which exhibits excellent performance [15].

Up to now, most studies on suspended graphene are realized via pristine graphene prepared by the mechanical exfoliation method [4]. Although the as-prepared pristine graphene is of the best quality so far with very high in-plane stiffness and high breaking strength, it is still quite limited for practical applications due to its relatively small size, ultra-low production, and high cost [4,16]. It is known that chemical vapor deposition (CVD) can produce large-area and high-quality graphene film [17,18,19]. One can imagine that suspended graphene film using the CVD method could combine the advantages of suspended graphene and CVD graphene, and hence have great potential for various applications, such as piezoresistive pressure sensors [20], mass sensing and spectroscopy [21,22], microphones [15,23,24], and temperature sensors [25]. However, recent research results indicate that the mechanical properties of graphene films prepared by CVD are much lower than those of mechanically exfoliated graphene [26,27,28,29]. For example, the failure strength was 45.4 ± 10.4 Gpa for CVD grain-free graphene films and 16.4 ± 5.1 GPa for CVD polycrystalline graphene films, which are significantly lower than that of 130 GPa for mechanically exfoliated graphene [30]. Therefore, improving the mechanical strength of CVD-grown graphene film is of great significance for using suspended CVD-grown graphene film in practical applications. Many efforts have been made to improve the mechanical properties of CVD-grown graphene film. Enzheng et al. dispersed carbon nanotubes on Cu foil, and then grew graphene in the carbon nanotube network by CVD to obtain carbon nanotube/graphene composite film [31]. This approach improves the mechanical strength by bonding graphene and carbon nanotubes, but also introduces new defects, particularly where carbon nanotubes are bonded to graphene. Khan et al. coated Polyethyleneimine (PEI) onto CVD Cu-based graphene and then etched the Cu foil to obtain a polymer composite film with good mechanical properties [32]. However, the mechanical properties of the polymer composite film depend on the polymer and not the graphene itself. Ruiz-Vargas et al. demonstrated by nanoindentation tests that the combination of two monolayer graphene films can form films with higher mechanical properties [27]. The stacked multilayer graphene can withstand greater loads before fracture [33], while still exhibiting the inherent properties of graphene film. Therefore, it is necessary to systematically study the effect of layer-by-layer CVD graphene film on the mechanical properties of suspended graphene film.

In this work, various suspended graphene films prepared by the CVD method were successfully obtained, including CVD-grown monolayer and multilayer graphene films, and stacked multilayer graphene films. By systematically studying the mechanical properties of as-prepared graphene film, the optimal suspended graphene film is selected and the corresponding mechanism is discussed. The result is very useful for further building high-performance suspended graphene-based electrical devices.

## 2. Materials and Methods

### 2.1. CVD Synthesis of Graphene Films

The graphene films are synthesized by the CVD method. Cu foil (Alfa Aesar, 20 μm thick, 99.9% purity) was heated to 1035 °C in an atmosphere of 100 standard cubic centimeters per minute (sccm) H_2_ and 500 sccm Ar at low pressure of 400 Pa. After reaching 1035 °C for 10 min, 30 sccm CH_4_ was introduced for 15 min to grow monolayer graphene on the surface of the Cu foil. Finally, the Cu foil with graphene on the surface was cooled to room temperature in an atmosphere of 10 sccm H_2_ and 500 sccm Ar. By increasing the growing pressure to 2000 Pa and extending the growth time to 20 min, three-layer graphene film was prepared. Six-layer graphene was obtained by introducing 20 sccm CH_4_ and 100 sccm H_2_ under atmospheric pressure for 10 min.

### 2.2. Stacking Graphene Film Layer-by-Layer Using Thermal Release Tape

A 4′4cm^2^ Cu-based graphene is laminated to the sticky side of the thermal release tape and the other side of the Cu-based graphene was removed using plasma. The samples were then etched in ammonium persulphate ((NH_4_)_2_S_2_O_8_) solution (0.5 mol/L) to etch the Cu. When the etching process was completed, the sample was slowly immersed in deionized water to remove any residual etchant and finally blown dry with nitrogen gas. The obtained thermal release tape/graphene was laminated onto the new Cu-based graphene to form a thermal release tape/graphene/graphene/Cu structure, and the sample was then heated to 140 °C to make the thermal release tape lose its adhesion, at which point a graphene/graphene/Cu structure was formed, thus completing the stacking of the two graphene films (Figure S1a–e). The lamination and adhesion release process of the thermal release tape/graphene and graphene/graphene/Cu was repeated to prepare stacked graphene with different layers.

### 2.3. Preparation of Suspended Graphene

The ultraviolet (UV) lithography process was used to fabricate circular hole arrays with different diameters on Cu foil with graphene films on top. The Cu foil with graphene films on top was firstly spin-coated with a photoresist. After spin coating, the first soft baking was carried out on the dryer to volatilize the solvent and enhance the adhesion between the photoresist and the substrate. The first exposure was carried out after the first soft baking sample cooled down. After the exposure was complete, a second baking was carried out to allow the first exposure area to form a mask, and then the second exposure was carried out this time without a mask plate (Figure S1f,g). At this point, the protective layer on the front of the sample has been prepared. After that, the same lithography procedure is carried out on the back of the sample with the exception that a mask plate is added during the first exposure.

After completing UV lithography on both sides of the sample, the sample was developed in the developer and then washed in deionized water to remove any residual developer. After blow-drying the sample with nitrogen gas, the template with circular hole arrays of different apertures was prepared. The sample was placed in (NH_4_)_2_S_2_O_8_ solution (0.5 mol/L) to etch the Cu. When the Cu in the hole array was etched, the sample was placed in deionized water to remove any residual etchant. After natural drying, the sample was placed positively down in acetone to remove photoresist from the graphene surface (Figure S1h–j).

### 2.4. Characterization

Surface morphology and mechanical properties of suspended graphene film were characterized by Raman spectroscopy (Renishaw inVia Reflex with laser excitation energy of 532 nm, Renishaw, London, UK), cold field emission scanning electron microscopy (SEM, S4800, 4 kV, Hitachi, Tokyo, Japan), polarized optical microscopy (POM, BX51, OLYMPUS, Tokyo, Japan), scanning probe microscope (SPM, Bruker Dimension ICON Billerica, MA, USA).

## 3. Results and Discussion

**Figure 1:**
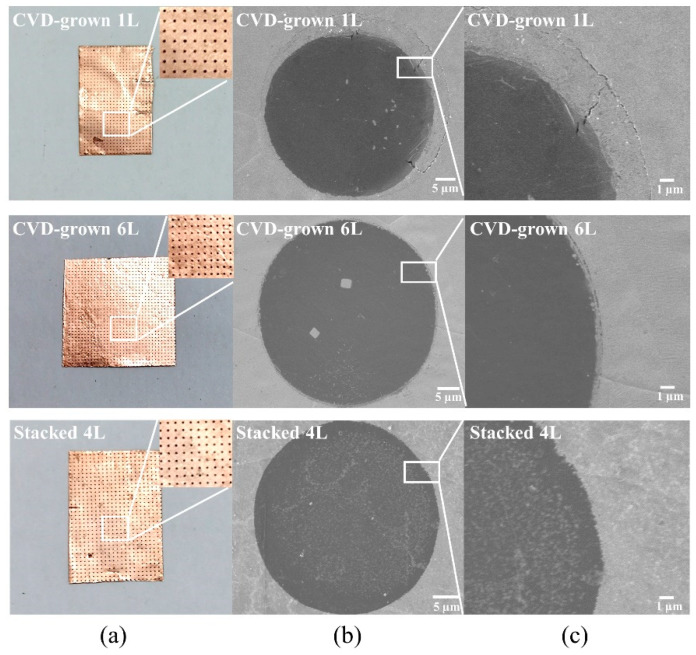
(**a**) Optical images of suspended graphene array with CVD-grown 1L, CVD-grown 6L, and stacked 4L. (**b**) SEM images of suspended graphene film with a diameter of 50 µm for CVD-grown 1L, CVD-grown 6L, and stacked 4L, respectively. (**c**) SEM images in larger magnification show the detailed edges of suspended graphene film.

The optical image of the suspended graphene array is shown in Figure 1a. After Cu etching, the suspended graphene array can already be seen, while the Cu foil remains intact. Figure 1b,c present SEM images of suspended graphene with different numbers of layers and different preparation methods as well as a plot of magnification. The CVD-grown monolayer (1L) graphene can be successfully suspended over circular holes with a diameter of 50 µm. However, it can be seen that the corresponding film is in a poor state. There are many small cracks in the film at the edge of the pores. In comparison, the CVD-grown 6-layer (6L) graphene is in excellent condition without cracks on the whole area over the circular hole with a diameter of 50 µm. Similarly, the stacked 4-layer (4L) graphene film can be well suspended over the whole suspended area. However, during the stacking process, more small impurity residues can be observed on the surface of the stacked 4L graphene film. By checking the surface morphology of the graphene after stacking in Figure S2, it was observed that there were few residual particles of thermal release adhesive. The residue was in diameter of less than 0.5 µm, and the particles existed independently of each other which would not form a connected network or film.

**Figure 2:**
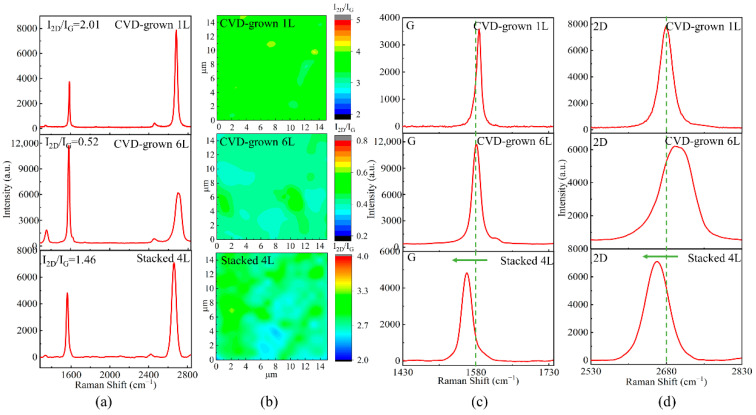
(**a**) Raman spectra for CVD-grown 1L, CVD-grown 6L, and stacked 4L, respectively. (**b**) Raman mapping of the ratio *I*_2_*_D_/I_G_*. (**c**) Enlarged G peaks in the Raman spectra and (**d**) enlarged 2D peaks in the Raman spectra.

To further characterize the structure of as-prepared suspended graphene film, Raman spectra are measured in detail as shown in Figure 2. The top Raman spectral curve in Figure 2a shows a high ratio of the 2D peak to the G peak intensity (*I*_2*D*_*/I_G_*) peak ratio of ~2 which demonstrates the nature of monolayer graphene, and the D peak on this curve is negligible, which demonstrates that the corresponding graphene film is low-defect monolayer graphene [34,35,36,37]. The middle Raman spectral curve in Figure 2a shows a low *I*_2*D*_*/I_G_* peak ratio of ~0.5, which demonstrates the characteristics of multilayer graphene [38]. By further measuring the light transmission of ~85.4%, the number of layers of the graphene film could be calculated as six on average. A strong D peak can be also observed, which might be due to the stitching misalignment of the grown grain boundaries of the multilayer graphene. This reveals the high defects in multilayer graphene films. The stacked 4L suspended graphene at the bottom of Figure 2a still fits the characteristics of monolayer graphene [39,40]. Even when stacked in multiple layers, the original properties of each graphene layer remain unchanged. Figure 2b shows the Raman mapping of the ratio *I*_2*D*_*/I_G_*. These results show that the prepared graphene films have a uniform number of layers, meanwhile, Figure S3f shows the prepared graphene crystal domains averaging at 20 µm.

Figure 2c,d show further comparison of the Raman G peaks and 2D peaks in detail. It can be observed that the G peak of the monolayer graphene film is located at 1587 cm^−1^, moisture and oxygen in the storage environment, as well as doping of the etchant, resulted in a slight shift from the typical G peak of other reported monolayer graphene [41,42]. Similarly, it can be observed that the 2D peak of the CVD-grown 1L graphene is located at 2680 cm^−1^ without any shift. In comparison, the Raman 2D peak of the CVD-grown 6L graphene film is located at 2696 cm^−1^ while the D peak is located at 1580 cm^−1^. The 2D peak position is shifted by 16 cm^−1^ towards the higher wave number, which is due to the splitting of the electronic band structure of multilayer graphene. It allows the 2D peaks to be fitted as the superposition of multiple Lorentz peaks, while the half-peak width of the 2D peaks gradually increases and shifts towards higher wave numbers as the number of layers increases [43,44]. However, the G peak level of the stacked 4L is shifted by 18 cm^−1^ towards the lower wave number, and the 2D peak level is shifted by 19 cm^−1^ towards the lower wave number [45]. This phenomenon indicates the presence of residual stresses in stacked suspended graphene. Some part of the residual stresses is due to stress stretching during the preparation of suspended graphene, while some part is due to stacking architecture. When graphene is under tensile strain, the carbon-carbon bonds are stretched and the Raman peaks are shifted towards lower wave numbers, in addition, when graphene is under compressive strain, the distance among the carbon atoms is reduced and the Raman peaks are shifted towards higher wave numbers. When graphene film is under a tensile strain of about 1%, a band gap of about 300 meV will be generated [45]. Therefore, the band gap of graphene can be adjusted by controlling the amount of tensile strain introduced during the stacking process, leading to the wider application of suspended graphene in electrical devices.

**Figure 3:**
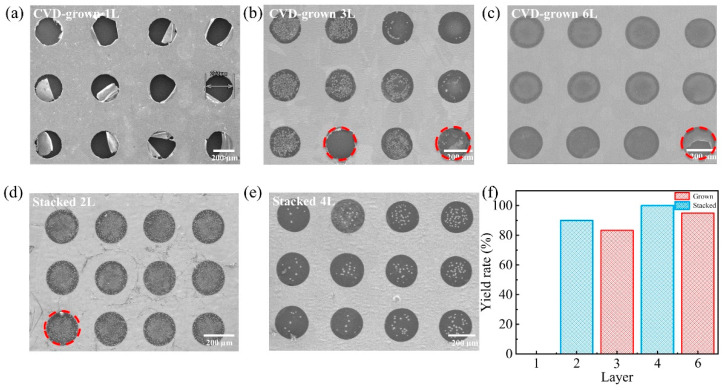
SEM images of (**a**) CVD-grown 1L graphene, (**b**) CVD-grown 3L graphene, (**c**) CVD-grown 6L graphene, (**d**) stacked 2L graphene film, (**e**) stacked 4L graphene film suspended on the circular holes in diameter of 200 µm. (**f**) Summary of the survival rate of suspended graphene film on the circular holes with a diameter of 200 µm with different layers and using different methods.

Figure 3 compares the SEM images of CVD-grown 1L, 3-layer (3L) and 6L versus stacked 2-layer (2L) and 4L suspended on the holes with a diameter of 200 µm. The survival rates are summarized in Figure 3f. As shown in Figure 3a, all monolayer graphene films were broken during fabrication and some of the broken graphene remained in the holes with a hole diameter of 200 µm. The phenomena might be caused due to the weak mechanical strength of monolayer graphene film which is unable to survive during the drying process [46]. This problem can be improved by increasing the layers of CVD-grown graphene film. As shown in Figure 3b,c, the survival rate of CVD-grown 3L graphene film reaches 83.3% and that of CVD-grown 6L graphene film reaches 90%, which is significantly higher than that of the monolayer graphene film. As observed in Figure S4a,b, although the CVD-grown 3L graphene film was able to survive, the liquid surface tension during the wet etching and photoresist removal process resulted in small cracks in the CVD-grown 3L graphene film. In contrast, the CVD-grown 6L graphene film does not have small cracks.

The size of the crystal domains can significantly affect the survival rate of suspended graphene [47]. The average size of the graphene crystal domain prepared in this experiment is 20 μm in Figure S3f. It can be predicted that at a minimum pore size of 50 μm, suspended graphene remains a polycrystalline film. As the size of suspended graphene increases, grain boundaries increase, which will lead to lower survival rates. During the growth process of multilayer graphene, the crystal domains of different layers grow at different rates, so when the surface domain is completely spliced, the bottom domain may still be unspliced, thereby limiting the survival rate of multilayer graphene. By increasing the size of the prepared graphene’s crystal domains, the grain boundaries can be reduced, thus increasing the survival rate of the suspended graphene on the micropores.

On the other hand, stacking multilayer graphene is another method to improve the survival rate. Figure 3d shows SEM image of stacked 2L graphene film suspended on circular holes in the same diameter of 200 µm. Compared to that the success rate of suspended monolayer graphene is 0%, that of stacked 2L graphene film reached 90% by simply adding one more monolayer graphene. Furthermore, the survival rate of stacked 4L graphene film reaches 100%, which is even higher than that of CVD-grown 6L graphene film. It suggests that layer-by-layer stacking might be a more effective means of increasing the survival rate of suspended graphene film.

**Figure 4:**
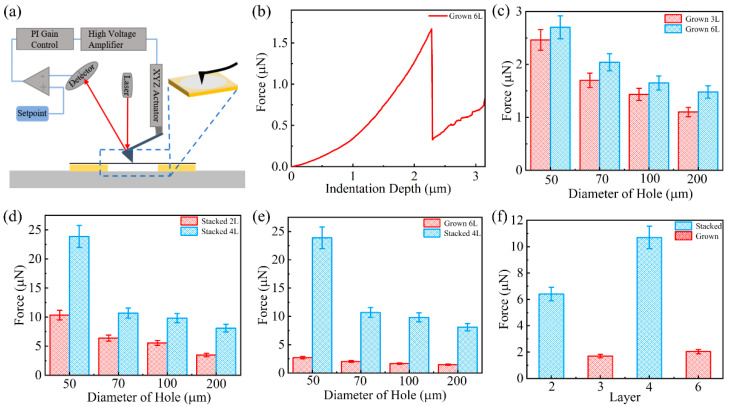
(**a**) Scheme of nanoindentation test on the suspended graphene film using AFM; (**b**) nanoindentation force vs. displacement curve measured on a typical CVD-grown suspended graphene with a diameter of 200 µm. (**c**) Comparison of the nanoindentation test results between the CVD-grown 3L and the CVD-grown 6L. (**d**) Comparison of the nanoindentation test results between the stacked 2L and the stacked 4L. (**e**) Comparison of nanoindentation test results with CVD-grown 6L versus stacked 4L. (**f**) Comparison of nanoindentation test results between CVD-grown graphene film and stacked graphene film with different layers suspended on holes with a diameter of 70 µm.

In order to quantitatively study the mechanical properties of suspended graphene film, nanoindentation experiments were carried out on various suspended graphene films with different layers using atomic force microscopy (AFM) [48,49]. The principle and test model for AFM nanoindentation test is illustrated in Figure 4a. Typically, point load is applied to the center of a suspended film using an AFM probe when the film has been deformed by the force. The flexural deformation data and the normal elasticity data of the film are measured to obtain the force-displacement curve. Firstly, the integrity of the sample films was observed using SEM before the AFM test was performed. Then, AFM testing was performed using the contact mode. For each sample, 15 intact suspended graphene films were randomly selected for test. Finally, the rupture of the sample films after the AFM test was observed using SEM after completing the AFM test to ensure the reliability of the AFM test data. Figure 4b presents a typical force-displacement curve of the nanoindentation test on a CVD-grown 6L suspended graphene with a diameter of 200 µm. From the zero position, the AFM probe starts to apply a point load on the suspended graphene film. As the load was applied, the film was gradually stretched. When the load was gradually increased to 1.67 μN, the film failed and the mechanical response weakened, then the load applied at the tip suddenly dropped. Figure S6a shows the suspended graphene film after failure, when the film was completely broken, cracks had already penetrated the whole film. The critical load that causes the film to break completely is considered to be the maximum force that the film can withstand, here the critical load is referred to as the fracture force. In addition, nanoindentation tests were performed on suspended graphene films in a randomly selected sample area, their breaking forces were collected, and summarized in the histogram.

The summarized results show that the maximum rupture force for a CVD-grown 3L suspended graphene with a diameter of 50 µm reaches 2.46 µN, while the one drops to 1.1 µN when the hole diameter is increased to 200 µm, as shown in Figure 4c. Similar trends can be observed in the case of the CVD-grown 6L, where the maximum fracture force of suspended graphene film decreases from 2.7 µN to 1.47 µN as the diameter of holes increases from 50 µm to 200 µm. It can be concluded that the mechanical properties of suspended graphene film become progressively worse as the diameter of holes increases. This phenomenon might be due to two reasons. Firstly, as the diameter of suspended graphene increases, defects in CVD-grown graphene gradually accumulate in probability, thus significantly affecting the mechanical properties of suspended graphene [28,50,51]. Secondly, as the diameter of the suspended graphene film increases, the surface tension of the solution that the graphene overcomes during the preparation of the suspended graphene also increases, leading to fine cracks in the graphene film [47,52]. This eventually leads to premature failure of the film, which affects the mechanical properties of the suspended graphene film.

Furthermore, it can be observed in Figure 4c, the mechanical properties of the CVD-grown 6L graphene film are approximately 20% higher than those of the CVD-grown 3L graphene film at a diameter of 70 µm. It is worth mentioning that the mechanical properties of CVD-grown graphene film are enhanced as the number of layers increases, but it does not show a doubled increase with doubling the layer number. This might be due to the defects in the CVD preparation of multilayer graphene. In Figure 2a, the Raman D peak intensity is higher for the CVD-grown 6L graphene film than for the CVD-grown 1L graphene film, indicating that there are more defects in the CVD-grown 6L graphene film and that these defects limit the improvement of mechanical properties of CVD-grown multilayer graphene films.

The nanoindentation tests are also performed on the suspended stacked graphene film, as shown in Figure 4d. The fracture force of suspended stacked 2L graphene film reaches 10.35 µN with a hole diameter of 50 µm, while it drops to 3.48 µN for the one with a hole diameter of 200 µm. The suspended stacked 4L graphene film reveals a very high fracture force of 23.87 µN with a hole diameter of 50 µm and still presents a fracture force of 8.1 µN with a hole diameter of 200 µm. By comparing the above results, it is found that the fracture force of suspended stacked 4L graphene film is increased by 130% compared to that of suspended stacked 2L graphene film. This demonstrates that doubling the number of layers in stacking results in the multiplication of mechanical properties, which is more effective than that of the direct CVD growth of multilayer graphene film as mentioned above. Comparing the nanoindentation test results of suspended CVD-grown 6L graphene film and suspended stacked 4L graphene film in Figure 4e, at 200 µm diameter, the fracture force of a stacked 4L graphene film is five times higher than that of a CVD-grown 6L graphene film. A comparison of nanoindentation tests of suspended graphene film prepared by different methods with the same hole diameter of 70 µm is shown in Figure 4f. It can be seen that the mechanical properties of the stacked 4L graphene film are improved by up to 400% compared to the CVD-grown 6L graphene film. It is demonstrated that stacking multilayer graphene films is a much more effective way to improve the mechanical properties of suspended graphene film, compared to the method of increasing the number of graphene layers through the CVD process.

**Figure 5:**
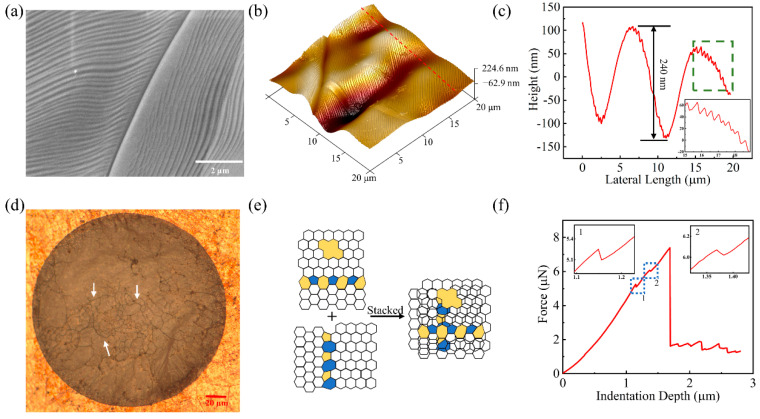
(**a**) SEM image, (**b**) AFM 3D image and (**c**) AFM line profile of the surface of a Cu foil with CVD-grown monolayer graphene film on top. Inset in (**c**) is an enlarged view of the dashed area. (**d**) Optical microscope image of stacked suspended graphene. (**e**) Scheme of stacking graphene film with defects between different layers. (**f**) Typical nanoindentation fracture curves of suspended stacked graphene film, and the inset is an enlarged view of the dashed region.

To further understand the phenomena observed above, the architecture of CVD-grown graphene film and the corresponding stacked multilayer graphene film is studied in detail. The SEM image in Figure 5a shows the surface morphology of the Cu foil with many fine stripes, grain boundaries and wave-like undulations after growing monolayer graphene film. The detailed surface roughness can be confirmed by the AFM 3D map in Figure 5b. The overall unevenness of the Cu foil ranges from −245 nm to 120 nm. This large roughness is caused by the Cu grain boundaries and the original rolling defects. A section of the selected test area was intercepted to observe the exact flatness of the Cu foil as shown in Figure 5c. The roughness profile shows large undulation on the surface of the Cu foil with a height variation of 240 nm and a height range of about 10–20 nm for the fine stripes. The graphene grown on the Cu substrate follows the surface morphology of the Cu foil as shown in Figure S7a. Thus, these undulations and steps on the surface of the Cu foil affect the flatness of the CVD-grown graphene film.

The graphene on the uneven part of the Cu foil will become folds after the etching process. These new folds are stacked on the surface of the film at the same time. When the load is loaded onto a suspended CVD-grown graphene film, the fold would be stretched and the film is then stretched. When the load is removed, the folds are in a post-stretch state, which could not recover its original tension and hence greatly affects the application of graphene film in devices.

Folds existing in the stacked graphene film can greatly enhance the mechanical properties of the entire film. During the stacking graphene layers process, the folds on each layer are also maintained. Folds in different layers can intersect each other to form a network. A network of fork-like folds can be clearly observed on the suspended stacked 4L graphene film in Figure 5d, as indicated by white arrows. In contrast, a similar network of fork-like folds is not observed on the CVD-grown 6L suspended graphene film in Figure S7b. Under the nanoindentation test, these forked folds are not on the same layer and they can support by the upper graphene film, preventing the spread of the folds and thus maintaining the original tension of the stacked graphene film on the prefabricated device configuration. These fork-like folds in the stacked film act as the backbone of the entire film, thus enhancing the mechanical properties of the entire film.

In contrast, polycrystalline multilayer graphene films prepared by CVD have line defects and point defects distributed throughout the whole surface. When a load is applied to the film, cracks are easily initialized at the defect. Once a crack has formed, it can expand rapidly and lead to the break of the entire film. As illustrated in Figure 5e, when two layers of graphene are stacked, the probability of two defects appearing in the same location is very small. Although graphene film may also develop cracks during the transfer stacking process, these cracks will also be covered by the upper layer of intact graphene, as demonstrated in Figure S8. Different layers of graphene films support each other to compensate for each other’s defects, which improves the mechanical properties of the stacked graphene film. Figure 5f shows a typical force-displacement curve for stacked graphene film. Firstly, one layer of the stacked film ruptures when the force reaches 5.25 µN. The fracture releases the accumulated stress through the indentation and the loading force suddenly becomes less, hence leading to the formation of a step in the curve. As the stress is released, the other layers of the stacked graphene film continue to support the whole film. As the force is further loaded to 6.07 µN, another layer of graphene film is broken, but there is still one layer of graphene to support the whole film until the force loading reached 7.4 µN. This curve demonstrates that stacked graphene layers support each other, thus enhancing the mechanical properties of stacked suspended graphene film.

## 4. Conclusions

In summary, suspended graphene film prepared by the CVD method, including CVD-grown monolayer and multilayer graphene film and stacked multilayer graphene film, were successfully prepared. As shown in the nanoindentation test results, at 70 µm diameter, CVD-grown multilayer graphene films only improve mechanical properties by 20%, while stacked multilayer suspended graphene films can improve them by 400%. Compared to the CVD-grown multilayer graphene directly, layer-by-layer stacking can compensate for defects in monolayer graphene and greatly reduce the probability of defect-induced rupture of suspended graphene. In addition, the inevitable folds generated from the folded dendritic network when stacking CVD-grown graphene film can further enhance the mechanical properties of the stacked suspended graphene. This work significantly improves the mechanical properties of CVD-grown suspended graphene film, which is important for expanding the electrical applications of CVD-grown suspended graphene film.

## Data Availability

All data that support the findings of this study are included within the article (and any supplementary files).

## Supplementary Materials

The following supporting information can be downloaded at: https://www.mdpi.com/article/10.3390/mi14040745/s1, Figure S1. Schematic diagram of the experimental process; Figure S2. Thermal release tape transfer graphene surface morphology. Figure S3. (a)–(d) Raman mapping of CV-grown graphene. (e) SEM of CVD-grown 3L and 6L graphene films on Cu foil. (f) Optical micrographs of graphene crystal domains in size. (g) Transparency comparison of CVD-grown graphene films. (h) sheet resistances of graphene films, which were averaged from 10 randomly selected places on each film. Figure S4. (a) CVD-grown 3L suspended graphene, with cracks indicated by red arrows. (b) CVD-grown 6L suspended graphene without cracks. Figure S5. (a) The state of the back of the hole in the etch. (b) Morphology of a fully ruptured graphene hole. (c,d) Enlarged view of the red dashed box in Figure (b). Figure S6. (a) The state of the film after failure following nanoindentation testing. (b–e) Results of nanoindentation tests for CVD-grown 3L, 6L and stacked 2L,4L. (f) Comparison of nanoindentation test results for stacked 2L and CVD-grown 3L. Figure S7. (a) Schematic diagram of graphene growth distribution on the surface of Cu ladder. (b) Growth of suspended graphene without stacked folds. Figure S8. (a) Schematic diagram of graphene crack stacking. (b) Schematic diagram of graphene fold stacking.
