# Supplementary material for: Significant Enhanced Mechanical Properties of Suspended Graphene Film by Stacking Multilayer CVD Graphene Films
Source: Micromachines (Basel). 2023 Mar 28;14(4):745. doi: 10.3390/mi14040745 (PMC10146136; doi:10.3390/mi14040745)
Supplement: Supplementary file 1 [file micromachines-14-00745-s001.zip › micromachines-2273664-supplementary.pdf]

Supporting Information

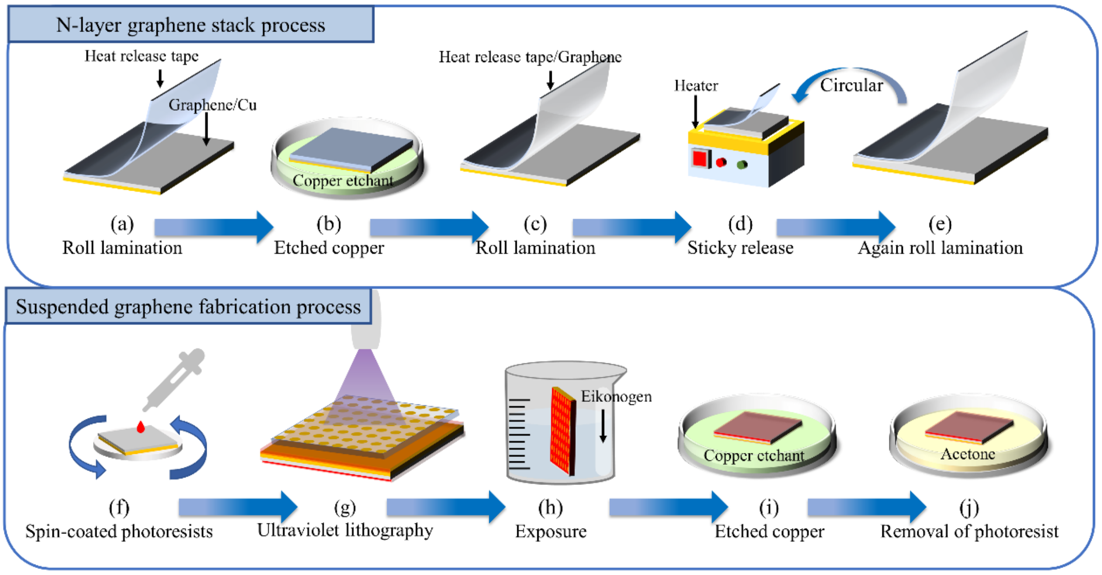

Figure. S1 Schematic diagram of the experimental process.

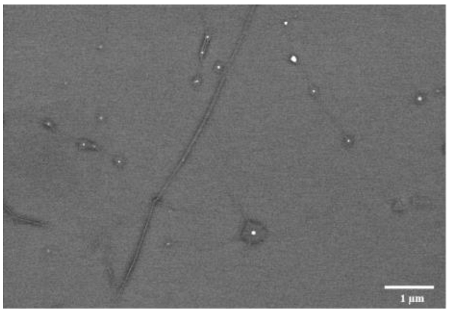

Figure. S2 Thermal release tape transfer graphene surface morphology.

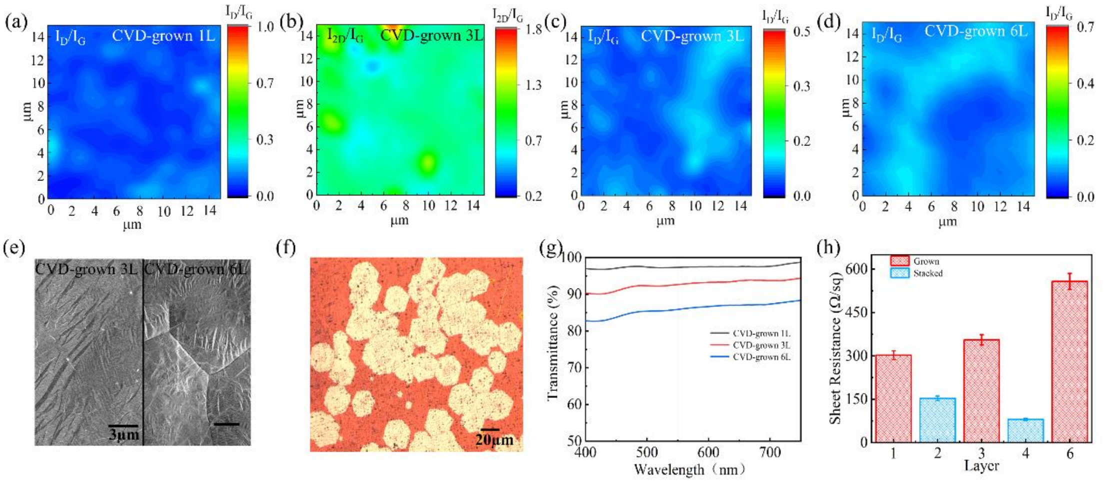

Figure. S3 (a)-(d) Raman mapping of CV-grown graphene. (e) SEM of CVD-grown 3L and 6L graphene films on Cu foil. (f) Optical micrographs of graphene crystal domains in size. (g) Transparency comparison of CVD-grown graphene films. (h) sheet resistances of graphene films, which were averaged from 10 randomly selected places on each film.

Through the graphene Raman mapping, it can be seen that the prepared graphene film is uniform as a whole. But with the increase of the number of graphene layers, the ratio of ID/IG gradually decreases, and the defects of the graphene film increase. In the comparison of CVD-grown 3L and 6L Cu-based graphene surface topography in Figure S2e, it can be observed that there are more folds in multilayer graphene. Figure S2f shows the domain size of the prepared graphene. The prepared graphene domain size is an average of 20um, which is smaller than the size of the smallest suspended graphene film. Figure S2g shows the transparency comparison of CVD-grown graphene film with different number of CVD-grown layers. The optical transmittance of CVD-grown 1L graphene film was 97.3%, and those of CVD-grown 3L and CVD-grown 6L graphene film were 92.6% and 85.4%, respectively, which further proves the uniformity of the prepared graphene film. The defects of graphene are also reflected in the resistance Figure S2h, CVD graphene layers increase, its resistance is also increasing.

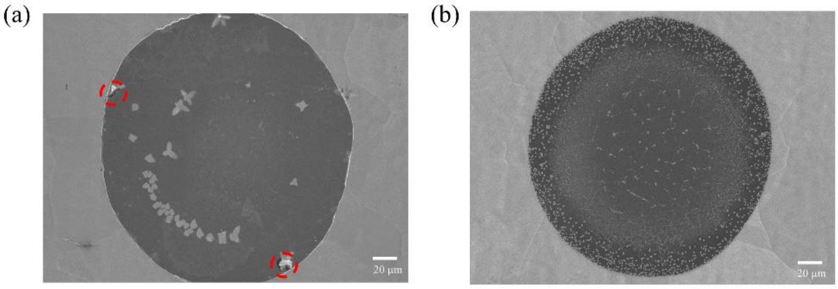

Figure. S4 (a) CVD-grown 3L suspended graphene, with cracks indicated by red arrows. (b) CVD-grown 6L suspended graphene without cracks.

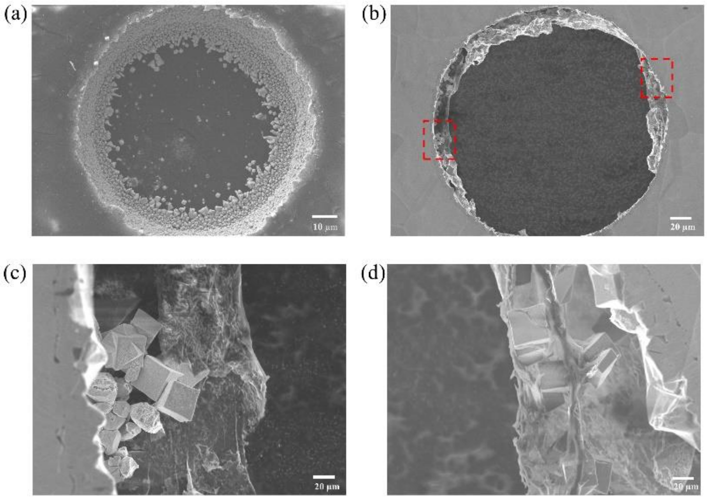

Figure. S5 (a) The state of the back of the hole in the etch. (b) Morphology of a fully ruptured

graphene hole. (c) and (d) Enlarged view of the red dashed box in Figure. b.

With careful observation, it is found that the holes in suspended graphene are not very regularly rounded, which may be due to the anisotropic etching during the wet etching process. Longitudinal etching and radial etching occur simultaneously during the etching process. To ensure a consistent concentration of etchant in the different holes during the etching process, uniform magnetic mechanical stirring is used. However, there is still a certain probability that radial etching and longitudinal etching will not achieve the intended target at the same time. Figure S4a illustrates the state of the suspended graphene backside during etching. It can be seen that in the current state, the radial etching has reached its target, but the longitudinal etching is still incomplete and the radial will be over-etched when the longitudinal etching is completed. At the same time, the presence of regularly shaped impurities on the graphene films was observed. These impurities are introduced during the Cu etching process, the production of Cu particles in etching can be seen in Figure S3. There is no way to stop the production of Cu particles during the etching process, and after rinsing the sample in deionized water three times after the etching is completed, there is still a certain probability of Cu particles falling on the graphene film during the final drying process.

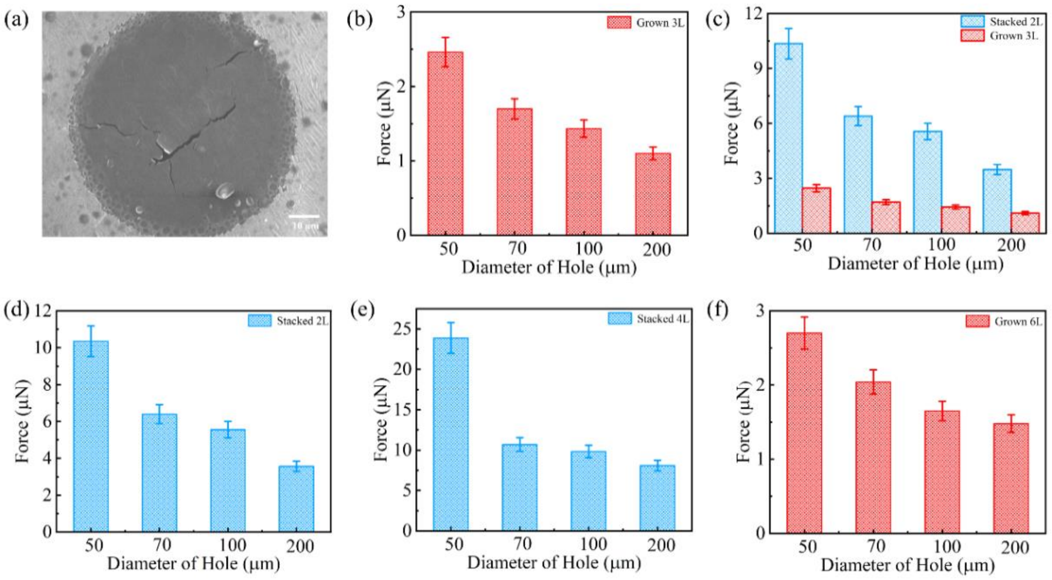

Figure. S6 (a) The state of the film after failure following nanoindentation testing. (b), (c), (d) and (e) Results of nanoindentation tests for CVD-grown 3L, 6L and stacked 2L,4L. (f) Comparison of nanoindentation test results for stacked 2L and CVD-grown 3L.

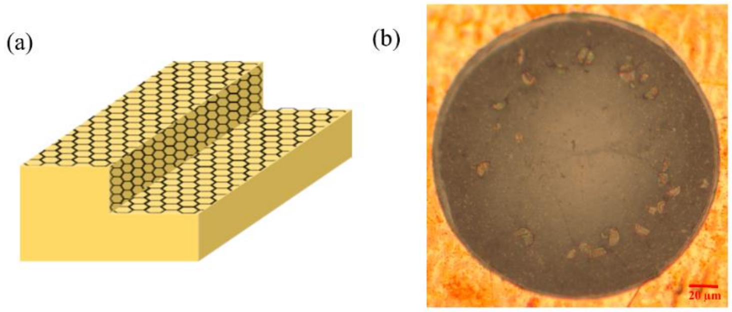

Figure. S7 (a) Schematic diagram of graphene growth distribution on the surface of Cu ladder. (b) Growth of suspended graphene without stacked folds.

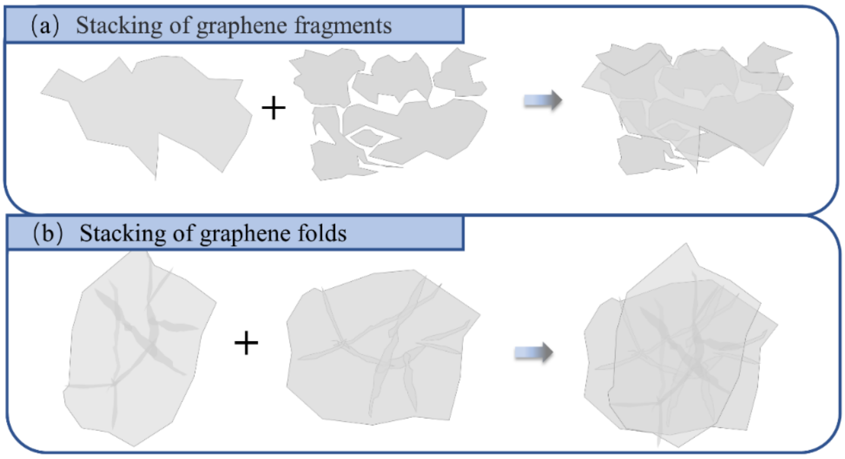

Figure. S8 (a) Schematic diagram of graphene crack stacking. (b) Schematic diagram of graphene fold stacking.
